# Evaluation of brain FDG PET images in temporal lobe epilepsy for lateralization of epileptogenic focus using data mining methods

**DOI:** 10.3906/sag-1911-71

**Published:** 2020-06-23

**Authors:** Ümit Özgür AKDEMİR, İrem YILDIRIM, Seda GÜLBAHAR, Kerim ŞEKER, Uğuray AYDOS, Gökhan KURT, Neşe İlgin KARABACAK, Lütfiye Özlem ATAY, Erhan BİLİR

**Affiliations:** 1 Department of Nuclear Medicine, Faculty of Medicine, Gazi University, Ankara Turkey; 2 Department of Neurology, Faculty of Medicine, Gazi University, Ankara Turkey; 3 Department of Neurosurgery, Faculty of Medicine, Gazi University, Ankara Turkey

**Keywords:** Temporal lobe epilepsy, F-18 fluorodeoxyglucose, positron emission tomography, data mining, classification

## Abstract

**Background/aim:**

In temporal lobe epilepsy (TLE), brain positron emission tomography (PET) performed with F-18 fluorodeoxyglucose (FDG) is commonly used for lateralization of the epileptogenic temporal lobe. In this study, we aimed to evaluate the success of quantitative analysis of brain FDG PET images using data mining methods in the lateralization of the epileptogenic temporal lobe.

**Materials and methods:**

Presurgical interictal brain FDG PET images of 49 adult mesial TLE patients with a minimum of 2 years of postsurgical follow-up and Engel I outcomes were retrospectively analyzed. Asymmetry indices were calculated from PET images from the mesial temporal lobe and its contiguous structures. The J48 and the logistic model tree (LMT) data mining algorithms were used to find classification rules for the lateralization of the epileptogenic temporal lobe. The classification results obtained by these rules were compared with the physicians’ visual readings and the findings of single-patient statistical parametric mapping (SPM) analyses in a test set of 18 patients. An additional 5-fold cross-validation was applied to the data to overcome the limitation of a relatively small sample size.

**Results:**

In the lateralization of 18 patients in the test set, J48 and LMT methods were successful in 16 (89%) and 17 (94%) patients, respectively. The visual consensus readings were correct in all patients and SPM results were correct in 16 patients. The 5-fold cross-validation method resulted in a mean correct lateralization ratio of 96% (47/49) for the LMT algorithm. This ratio was 88% (43 / 49) for the J48 algorithm.

**Conclusion:**

Lateralization of the epileptogenic temporal lobe with data mining methods using regional metabolic asymmetry values ​​obtained from interictal brain FDG PET images in mesial TLE patients is highly accurate. The application of data mining can contribute to the reader in the process of visual evaluation of FDG PET images of the brain.

## 1. Introduction

Brain F-18 fluorodeoxyglucose (FDG) positron emission tomography (PET) is a frequently used diagnostic imaging method for the evaluation of drug-resistant epilepsy patients [1]. In epilepsy, cortical hypometabolism is seen in the interictal period in the epileptogenic brain region, which is responsible for the onset of seizures, and in other related brain regions where epileptic activity spreads [1,2]. The mechanism underlying hypometabolism in epilepsy is believed to include neuronal loss, a decrease of synaptic density and diaschisis [2]. Hypometabolism observed in the ipsilateral temporal lobe is a finding that contributes to lateralization of epileptogenic focus in drug-resistant mesial temporal lobe epilepsy (TLE) patients undergoing surgical treatment [3-8]. The rate of ipsilateral temporal hypometabolism in TLE varies between 60% and 100% depending on the different analysis methods used and differences in patient groups in the studies [1]. This finding also has a significant prognostic impact regarding patient outcomes [1,5,7–12]. In a metaanalysis of 46 publications (between 1992 and 2006), it was shown that hypometabolism in the ipsilateral temporal lob in TLE patients had a predictive value of 86% for a good postsurgical outcome [4]. Predictive values of the same finding in TLE patients with normal magnetic resonance imaging (MRI) and nonlocalized ictal scalp electroencephalography (EEG) were 80% and 72%, respectively [4]. Therefore, brain FDG PET imaging may contribute to patient management, especially in cases where the patient’s brain MRI findings are normal and clinical findings are inconclusive [1,4,8,13].

In routine clinical practice, brain FDG PET images are often visually evaluated by physicians [2]. However, it has been shown in several studies that the use of quantitative analysis in the evaluation of brain FDG PET images in terms of localization and lateralization of epileptogenic focus increases the diagnostic accuracy of the examination [2,9,10,12,14]. Voxel or region-of-interest (ROI)-based quantitative analysis can be performed by statistically comparing the brain FDG PET image of the patient with a normal FDG PET database [1–3,6,10,11]. Another commonly used analytical approach is to determine regional metabolism values ​​and to investigate the presence of asymmetric involvement between homologous brain regions in the two hemispheres [2,3,5,11,15]. The advantage of ROI-based calculation of asymmetry over the voxel-based analysis is that the values ​​obtained from the PET images reflect relative differences in FDG uptake between homologous regions and therefore do not need a count normalization [2,9,15]. In several FDG PET studies performed in mesial TLE, the methods based on the calculation of asymmetry have shown better results than the voxel-based methods in terms of lateralization of the epileptogenic lobe, reaching accuracy values over 90% [3,5,6,9,11,16]. 

In mesial TLE, in addition to the ipsilateral mesial temporal lobe, hypometabolism may also be observed in its neighboring neocortical temporal regions, ipsilateral insula, frontal lobes, ipsilateral thalamic nucleus, and contralateral temporal lobe [3,6,10,16–18]. Mild compensatory increase of metabolism in the contralateral temporal lobe may also be observed [3,16,19]. Hypometabolism in remote (distant to and not contiguous with perifocal hypometabolism) cortical regions, which is probably involving seizure propagation pathways and indicating the effects of seizures on these networks, has prognostic implications in patients operated for TLE [6,7,17,18,20]. Patients with remote hypometabolism in extratemporal regions are reported to have worse postsurgical outcomes [6,7,17,18]. This extensive hypometabolism may complicate visual assessment and may be regarded as a limitation of brain FDG PET imaging for the localization of the epileptogenic region [12]. However, it may also contribute to the lateralization of epileptogenic temporal lobe if quantitative analysis is used [3,5,6,9,11,20]. 

The lateralization of the epileptogenic temporal lobe in patients with mesial TLE using brain FDG PET data is a problem of classification. In this respect, data mining methods may be applied to classify PET images as right- or left-sided TLE and the outputs of the classification algorithm may support the human interpreter with classification and differential diagnosis [21]. The application of data mining is based on the evaluation of quantitative data obtained from images by statistical methods, such as logistic regression, model trees, and naive Bayes classifiers [21–23]. In TLE patients with a favorable postsurgical outcome, the definite lateralization results may be used in the supervised learning of data mining methods. Then test data may be classified with the trained data mining algorithm. Although there are many studies in the literature using both voxel- and ROI-based numerical analyses for lateralization in TLE, to the best of our knowledge, there are only two studies in which data mining methods are applied to the brain FDG PET data [24,25]. In one of these studies Peter et al. showed that lateralization indices calculated from temporal regions are reliable and reproducible measures for predicting seizure lateralization in unilateral TLE patients [25]. In the second study, Lee et al. compared the performance of a computer-aided classifier using an artificial neural network with the reading performance of expert physicians in the lateralization of TLE and found an 85% average agreement [24]. The aforementioned studies suggest that data mining applications can provide supportive information for the lateralization of TLE. Therefore, the hypothesis of this study is that lateralization of the epileptogenic lobe in TLE as a classification problem can be performed with high accuracy using data mining methods and data obtained from the quantitative regional analysis of brain FDG PET images. This study aimed to develop a classification method using data mining methods for the lateralization of epileptogenic lobe in TLE patients which depends on the calculation of asymmetry indices from regional FDG PET data and to evaluate the accuracy of this method in comparison to visual reading performances and the voxel-based quantitative analysis. 

## 2. Materials and methods

### 2.1. Patient population

In this study, data of TLE patients who were surgically treated and had a presurgical diagnostic interictal brain FDG PET examination with the routine clinical indication of localization and lateralization of the epileptogenic focus were analyzed retrospectively. Adult patients (age > 18 years) who had successful treatment response (Engel I: “Free of disabling seizures”) in clinical follow-up of at least two years after surgical treatment were included in the study [26]. Therefore, the final clinical lateralization of TLE depended on the successful postsurgical outcome. The study was found to be ethically appropriate with the decision (numbered 20 and dated 14.01.2019) of the Gazi University Faculty of Medicine Clinical Research Ethics Committee.

### 2.2. Brain FDG PET imaging

The routine interictal brain FDG PET protocol applied in our center includes fasting of the patient for a minimum of 6 h before injection of 0.1 mCi/kg FDG. Throughout the FDG uptake period patient rests still in a dimly-lit, quiet room, with eyes open to minimize cerebral activation. Static brain PET images were acquired on a Discovery ST PET-CT camera (GE Medical Systems) 60 min after FDG injection. A computerized tomography (CT) scan for attenuation correction and a 15 min PET emission acquisition in the 3D mode were done. PET images were reconstructed using an iterative-reconstruction algorithm.

### 2.3. Quantitative analysis of PET images

The SPM8 (Wellcome Department of Cognitive Neurology, Institute of Neurology, University College London, London, UK) and the WFU PickAtlas (ANSIR Laboratory, Wake Forest University School of Medicine, Winston-Salem, NC) programs were used for quantitative evaluation of brain FDG PET images [27-29]. Spatial normalization process was applied to the brain FDG PET images by using the SPM8 program and the FDG PET template image which was created institutionally. In this way, patient images were placed in the standard Montreal Neurological Institute (MNI) space. All normalized images were visually checked for the success of normalization.

In order to obtain regional mean count values ​​from normalized PET images, the definitions of Automated Anatomical Labeling (AAL) atlas were used [30]. Thirteen regions (opercular part of inferior frontal gyrus, rolandic operculum, insula, hippocampus, parahippocampal gyrus, amygdala, supramarginal gyrus, thalamus, superior temporal gyrus, temporal pole of superior temporal gyrus, middle temporal gyrus, temporal pole of middle temporal gyrus, and inferior temporal gyrus) in the AAL brain atlas that were contiguous or functionally associated with mesial temporal lobe structures and reported to show hypometabolism in the studies on FDG PET imaging in mesial TLE were selected [3,6,16,18]. Regional asymmetry indices (AI) ​​for each selected area of ​​interest (ROI) were calculated using the formula: AIROI = (left hemisphereROI – right hemisphereROI) / (left hemisphereROI + right hemisphereROI) × 200.

### 2.4. Data mining process 

**Figure 1:**
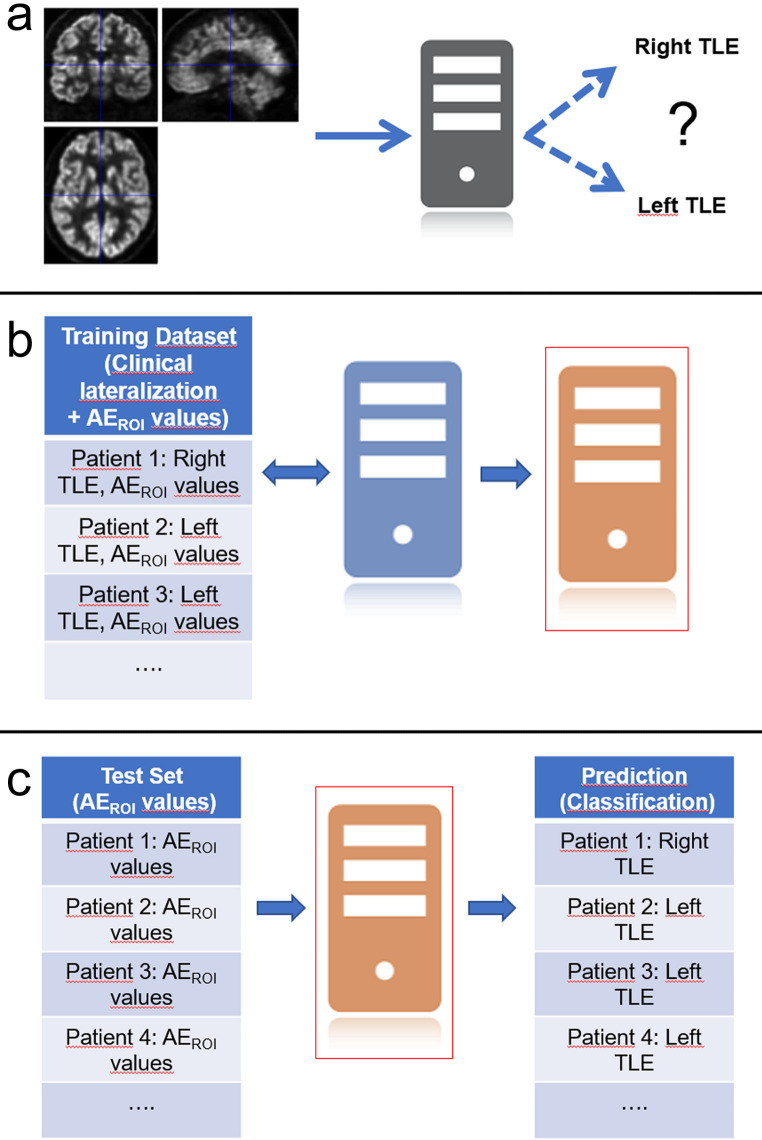
The data mining process. (a) The aim of the data mining process in this study is to lateralize interictal brain FDG PET images of patients with mesial TLE as “Right TLE” or “Left TLE”. (b) For this purpose, the classification method (represented by the blue computer) is provided with quantitative data (AIROI) obtained from the regional analysis of PET images together with the labels (definitive lateralization information) of the patients in the training set and will produce a set of classification rules (represented by the orange computer) in this supervised training process. (c) Then, the classification rules are used to make predictions from the similar quantitative data of the patients in the test set.

The 49 TLE patients included in the study were divided into training (n = 31, 63%) and test (n = 18, 37%) sets through randomization and by applying a customary proportion of approximately 60:40 [31]. In the R software, J48 (an entropy-based C4.5 algorithm) and LMT classification tools included in the RWeka package were used [22,23,32,33]. In a supervised learning session, lateralization models were generated by using J48 and LMT tools on the training set which included the final clinical lateralization data of patients and AIROI values ​​of 13 selected regions from the PET data (Figures 1a and 1b). Subsequently, the accuracy of the two models in terms of lateralization was evaluated using the test set (Figure 1c).

Since the total number of patients was relatively limited, a k-fold cross-validation method was used in the study [31]. For this purpose, the whole dataset of 49 patients was divided into five nonoverlapping sets by randomization. Five runs of training and testing were carried out for each of these five sets, by using one set as the test and the other four as the training sets. Then, the means of correct lateralization rates were calculated for J48 and LMT algorithms by averaging the ratios obtained in each run.

### 2.5. Visual assessment of PET images

Brain FDG PET images of 18 patients in the test set were anonymized and prepared for visual evaluation as gray-scale axial PET image slices in a standard anatomical orientation (axial plane parallel to the frontal-occipital line) and ordered sequentially from vertex to cerebellum. Two nuclear medicine physicians, one with eight and the other with four years of brain FDG PET reading experience, were asked to lateralize the epileptogenic temporal lobe of the patient. The physicians evaluated these images separately, and then they did a consensus reevaluation of inconsistent readings.

### 2.6. SPM analysis of PET images

SPM is a piece of software for statistical analysis of brain images at the voxel level and creating statistical parametric maps. In this program, using a statistical model (such as t-test, ANOVA) which is appropriate for the research question and the selected statistical and voxel extension thresholds, the brain regions that differ significantly are determined. The statistical threshold value (P) is decisive for statistical significance. The voxel extension threshold imposes a limitation on the neighborhood relationship of voxels exceeding the statistical threshold so that adjacent voxels exceeding the statistical threshold are only displayed if they are greater than the specified number.

PET images of the test set of patients (n=18), which were evaluated both by data mining methods and visually, were also evaluated by using SPM5. For this purpose, an institutional normal brain FDG PET database containing preprocessed and spatially normalized brain FDG PET images of 44 adult (mean age ± SD = 60.7 ± 10.6 years) patients were used. The SPM method has been validated as a tool to quantify hypometabolic patterns in a single patient’s brain FDG PET image [3,6,10–12,34]. Following the spatial normalization of PET image to the MNI template and a 10 mm isotropic Gaussian smoothing, each patient’s FDG PET image was tested for relative hypometabolism by comparison with the normal PET database on a voxel-by-voxel basis using the general linear model, employing the two-sample t-test design. Family-wise error (FWE) corrected statistical significance threshold of P = 0.005 at the voxel level and extend threshold of 250 voxels at the cluster level were used for the detection of hypometabolism. According to the result of the analysis performed with SPM5, if ipsilateral temporal hypometabolism consistent with definite clinical lateralization was observed, it was evaluated as correct lateralization. If hypometabolism was observed in both temporal lobes, the success of lateralization was decided by considering the side with the most extensive temporal lobe involvement.

### 2.7. Statistical analysis

Continuous variables are expressed as mean ± standard deviation (SD). Cohen’s kappa statistics were used to evaluate the results of the data mining algorithms and their compatibility with the exact clinical lateralization. The SPSS (version 23) statistical software was used for statistical analyses.

## 3. Results

Presurgical interictal brain FDG PET images of 49 adult patients (mean age ± SD = 36.2 ± 7.7) who underwent surgical treatment (selective amygdalahipocampectomy ± anterior temporal lobectomy) with the diagnosis of drug-resistant TLE and had a favorable outcome (Engel I) during postsurgical clinical follow-up were evaluated (Table S1). In the patient group, female-to-male ratio was 32/17, mean duration of epilepsy was 17.7 ± 7.7 years, and mean duration of postsurgical follow-up was 53.2 ± 28.7 months. According to the surgical and postsurgical clinical findings 22 patients had right TLE and 27 patients had left TLE as their final diagnoses.

**Figure 2:**
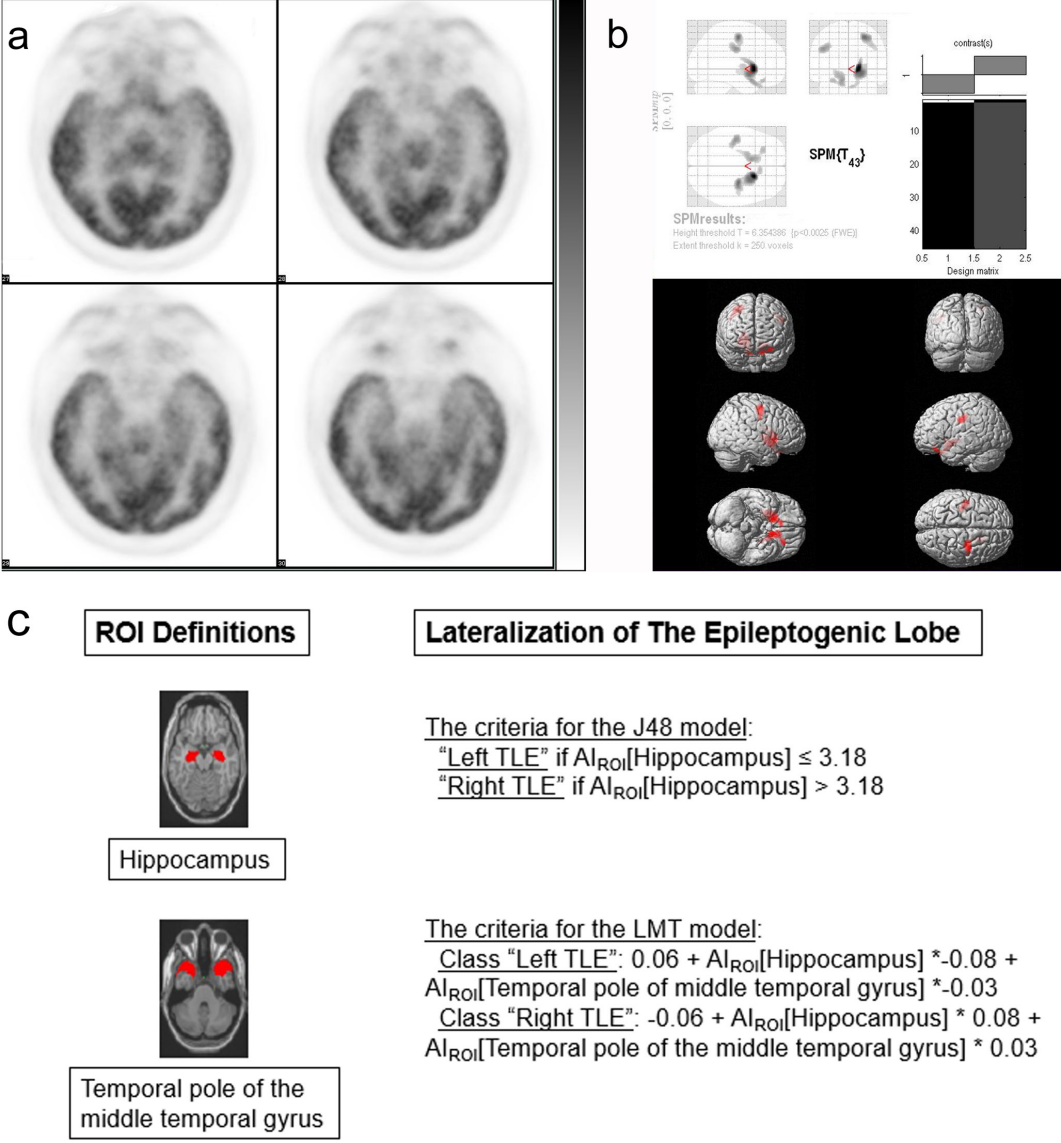
An example of a patient’s lateralization results of the three methods used in the study. The patient (The patient with number 12 in Table 1) presented here is a 37-year-old female with focal impaired awareness seizures and focal to bilateral tonic-clonic seizures which started at the age of 18. The patient was operated for left mesial TLE and had Engel IA postsurgical outcome in a follow-up period of 29 months. (a) The axial brain FDG PET slices that pass through the long axis of temporal lobes are shown. The visual reading of the patient’s FDG PET images with the consensus of two nuclear medicine physicians was “Left TLE”. (b) The SPM analysis of the patient’s PET data against the institutional normal brain FDG PET database showed no statistically significant hypometabolic clusters in the temporal lobes; therefore, it was accepted as “No lateralization”. (c) The criteria for lateralization obtained by the J48 and LMT algorithms and the relevant ROI definitions (red-colored regions) in the AAL brain atlas are shown. The patient’s AIROI values were 5.71 and 7.94 for the hippocampus and the temporal pole of the middle temporal gyrus respectively. According to these values both of the data mining methods falsely lateralized the patient’s PET data as “Right TLE” which was the single case of false lateralization of the LMT classification algorithm.

When the test set of 18 randomly selected patients were evaluated with the models obtained by J48 and LMT algorithms according to the training set of 31 patients, it was observed that the J48 model correctly lateralized 16 (89%) patients and the LMT model correctly lateralized 17 (94%) patients (Table 1). For the classification results obtained by J48 and LMT models, Cohen’s kappa values were 0.775 (T = 3.288, P = 0.001) and 0.889 (T = 3.795, P < 0.001), respectively. In the same test set of patients, the first reader (with four years of brain FDG PET reading experience) correctly lateralized all patients and the other reader falsely lateralized one patient. In their consensus reading, they correctly lateralized all patients (Figure 2a). SPM analysis of the test set produced no false lateralizations. However, in two patients SPM analysis showed no temporal hypometabolism and did not produce any lateralization information (Figure 2b). 

**Table 1:** The lateralizations of TLE patients in the test group (n = 18) by visual assessment of nuclear medicine physicians, SPM analysis, and data mining methods (J48 and LMT algorithms) in comparison to the definitive lateralization according to the postsurgical favorable (Engel I) outcomes.

Test data (Patient numbers)	Definitive lateralization	Visual assessment			SPManalysis	J48algorithm	LMT algorithm
		Reader1	Reader2	Consensus			
1	Left	Left	Left	Left	Left	Left	Left
2	Left	Left	Left	Left	Left	Left	Left
3	Left	Left	Left	Left	Left	Left	Left
4	Left	Left	Left	Left	Left	Left	Left
5	Left	Left	Left	Left	Left	Left	Left
6	Left	Left	Left	Left	Left	Left	Left
7	Left	Left	Left	Left	Left	Left	Left
8	Left	Left	Left	Left	Left	Left	Left
9	Right	Right	Right	Right	Right	Right	Right
10	Right	Right	Right	Right	Right	Right	Right
11	Left	Left	Left	Left	Left	Left	Left
12	Left	Left	Left	Left	No lateralization	Right	Right
13	Right	Right	Left	Right	Right	Right	Right
14	Right	Right	Right	Right	No lateralization	Right	Right
15	Right	Right	Right	Right	Right	Right	Right
16	Right	Right	Right	Right	Right	Right	Right
17	Right	Right	Right	Right	Right	Right	Right
18	Right	Right	Right	Right	Right	Left	Right
Correct lateralization (ratio, %)		18/18, 100%	17/18, 94%	18/18, 100%	16/18, 89%	16/18, 89%	17/18, 94%

Note: Italic characters are used whenever the lateralization is not successful (“No lateralization”) or false in comparison to the definitive lateralization. Left and right refer to left TLE and right TLE, respectively. The criteria for the J48 model were “Left TLE” if AIROI[Hippocampus] was lower than or equal to 3.18 and “Right TLE” if AIROI[Hippocampus] was greater than 3.18. The criteria for the LMT algorithm were as follows: Class “Left TLE”: 0.06 + AIROI[Hippocampus] × –0.08 + AIROI[Temporal pole of middle temporal gyrus] × –0.03 and Class “Right TLE”: –0.06 + AIROI[Hippocampus] × 0.08 + AIROI[Temporal pole of the middle temporal gyrus] × 0.03.

In the application of k-runs cross-validation method with five nonoverlapping test sets, the mean correct lateralization rates were 43/49 (88%) for the J48 and 47/49 (96%) for the LMT algorithms (Table 2). The classification rules produced by the J48 algorithm in each run involved AI calculated from either inferior temporal gyrus, rolandic operculum, or parahippocampal gyrus. The classification rules produced by the LMT algorithm depended on AI calculated from the hippocampus and temporal pole of the middle temporal gyrus (Figure 2c).

**Table 2:** The results of 5-runs of data mining algorithms according to the k-fold cross-validation method using the randomly produced training and test sets.

k-fold cross-validation method	J48 algorithm		LMT algorithm	
	Model	Correct lateralization (ratio, %)	Model	Correct lateralization (ratio, %)
1st run	AIROI[Inferior temporal gyrus] ≤ –3.99: LeftAIROI[Inferior temporal gyrus] > –3.99: Right	9/10, 90%	Class Left : 0.13 + AIROI[Hippocampus] × –0.07 + AIROI[Temporal pole of middle temporal gyrus] × –0.02Class Right : –0.13 + AIROI[Hippocampus] × 0.07 + AIROI[Temporal pole of middle temporal gyrus] × 0.02	10/10, 100%
2nd run	AIROI[Inferior temporal gyrus] ≤ –3.99: LeftAIROI[Inferior temporal gyrus] > –3.99: Right	10/10, 100%	Class Left : 0.14 + AIROI[Hippocampus] × -0.07Class Right : –0.14 + AIROI[Hippocampus] × 0.07	9/10, 90%
3rd run	AIROI[Parahippocampal gyrus] ≤ –5.46: LeftAIROI[Parahippocampal gyrus] > –5.46: Right	9/9, 100%	Class Left : 0.16 + AIROI[Hippocampus] × –0.07 + AIROI[Temporal pole of middle temporal gyrus] × –0.02Class Right : –0.16 + AIROI[Hippocampus] × 0.07 + AIROI[Temporal pole of middle temporal gyrus] × 0.02	9/9, 100%
4th run	AIROI[Rolandic operculum] ≤ 2.06: LeftAIROI[Rolandic operculum] > 2.06: Right	8/10, 80%	Class Left : 0.09 + AIROI[Hippocampus] × –0.07 + AIROI[Temporal pole of middle temporal gyrus] × –0.03Class Right : –0.09 + AIROI[Hippocampus] × 0.07 + AIROI[Temporal pole of middle temporal gyrus] × 0.03	9/10, 90%
5th run	AIROI[Parahippocampal gyrus] ≤ –0.24: LeftAIROI[Parahippocampal gyrus] > –0.24: Right	7/10, 70%	Class Left : 0.23 + AIROI[Hippocampus] × –0.07 + AIROI[Temporal pole of middle temporal gyrus] × –0.02Class Right : –0.23 + AIROI[Hippocampus] × 0.07 + AIROI[Temporal pole of middle temporal gyrus] × 0.02	10/10, 100%
Mean		43/49, 88%		47/49, 96%

Note: The models show the classification criteria obtained in each run of J48 and LMT algorithms using the training set. For this purpose the whole study group is randomly divided into five smaller, nonoverlapping subgroups while trying to preserve a similar ratio of left-to-right TLE in each subgroup. The subgroups included 10 patients in all except one which included 9 patients. Then, a five-fold cross-validation procedure was applied by using one subgroup as the test set and the other subgroups all together as the training set in each run. The mean correct lateralization ratios were calculated for J48 and LMT algorithms.

## 4. Discussion

In this study, it was shown that the evaluation of interictal brain FDG PET images in mesial TLE patients with data mining methods has a high accuracy rate in terms of lateralization of the epileptogenic temporal lobe. Accurate clinical lateralization information based on postsurgical follow-up findings was used as a reference in the assessment of correct lateralization. LMT algorithm, which forms a classification model by using the asymmetry values of multiple regions together, has been observed to perform correct lateralization at a rate of 96% with the k-run cross-validation method conducted on the data of 49 patients. With the same approach, the lateralization success of the J48 algorithm was 88%. When the whole patient group was divided into learning and test sets with a customary ratio of 60:40, these two algorithms have high lateralization success close to the results of the visual evaluation and SPM analysis in the test set of 18 patients.

The use of data mining in the analysis of medical data is currently becoming a popular issue. Data mining methods are applied in order to use various data obtained from medical examinations in the diagnosis of diseases [21]. In this respect, data mining can be defined as the work of extracting implicit information of value among large-scale data [31]. In order to classify diseases using diagnostic data, it is necessary to create a model that reveals hidden patterns in these data sets. Entropy-based methods, logistic regression models, Bayesian classifiers, and artificial neural networks are the commonly used classification methods [21–23,31,33]. The classification rules that are formed by applying these methods on training data are then applied to test data in order to predict the class of each subject. The success of the classification of the model is evaluated by comparing these predictions with the actual clinical data. In this study, a successful example of data mining for lateralizing the epileptogenic temporal lobe by utilizing interictal brain FDG PET images in mesial TLE patients was performed. 

In the medical literature, there are few studies using data mining in epilepsy patients [24,25]. Peter et al., in their retrospective study on 17 TLE patients, showed that by using the lateralization index calculated from the temporal lobe alone and a machine learning logistic regression analysis approach it was possible to lateralize the epileptogenic temporal lobe with 82% accuracy [25]. However, the lateralization information in this study was based on the results of the diagnostic examination which also included the FDG PET study. In another study, the performance of a computer-assisted classifier using an artificial neural network in lateralization was compared with the reading performance of the experts in 261 TLE patients and an average 85% agreement was observed [24]. In our study, since the classification of the patients are confirmed by the favorable outcome after surgery, the diagnostic performance of artificial intelligence algorithms could be evaluated more reliably. 

Methods which are used to quantitatively evaluate brain metabolism can be divided into two categories according to the ROI used: The voxel-based analysis in which the ROI is defined as a single voxel and the ROI-based analysis in which the mean values ​​of multiple voxels within anatomically restricted regions are used [1,2]. The high number of voxels that make up the PET images of the brain necessitate the use of special software such as SPM with statistical corrections specific to multiple analysis in the voxel-based analysis approach [27]. SPM analysis can be used for statistical analysis of group data or to identify hypometabolic brain regions in a single patient data [12,34]. However, especially for the second type of use, a normal brain FDG PET database to compare the patient’s PET image is required [12,34]. Similarly, in the ROI-based approach, a normal database can be used to detect hypometabolism independent of regional asymmetry [9]. One of the most important steps of this approach is how the ROIs are defined. For standardization of ROI definitions and to improve the quantification of brain images, tools such as AAL which are prepared according to a common anatomical brain space definition (such as the MNI atlas) and contain spatial definitions of brain regions are generally used [28–30]. In this study, AAL definitions that are widely used in quantitative analysis of brain PET images and which can be run with WFU PickAtlas toolbox under the SPM were used [27–30].

There are many studies which evaluated the success of lateralization of epileptogenic lobe from interictal brain FDG PET images in TLE [3–6,9–12,20,35,36]. These studies generally show that PET examination has high diagnostic accuracy when evaluated quantitatively and the quantitative analysis contributes to the visual evaluation of the readers [5,11,12,35,36]. A metaanalysis study based on the data of 46 studies conducted between 1992 and 2006 showed that ipsilateral temporal hypometabolism predicted good postsurgical outcome in TLE patients, thus providing accurate lateralization information [4]. More recent studies suggest that PET imaging has a lateralization success greater than 90% in TLE [5,9]. The contribution of quantitative analysis increases especially in cases where the reader’s PET imaging experience is limited or there is an epileptogenic focus outside the temporal lobe [12,35,36]. In contrast to the subjective assessment of the reader, quantitative analysis is performed independently of the impact of the reader and provides objective findings. The normalization of the patient’s PET image to a standard anatomical atlas such as MNI and the use of standard ROI definitions defined eliminates possible erroneous approaches of the processor and bias that may arise in this regard. There is no direct confounding effect of the reader on the results during the quantitative analysis and data mining applications used in this study. However, it is the responsibility of the reader to optimize the standardization of the analytical method and to observe possible sources of error (such as patient movement, low image quality, incorrect spatial normalization) during the analysis.

In the visual evaluation process, the reader assesses the distribution of cortical metabolism, particularly investigating differences in involvement between homologous brain regions in the two hemispheres. In this regard, the evaluation of brain FDG PET examination may be considered a lateralization problem especially in TLE patients. In epilepsy, interictal brain FDG PET imaging shows signs of hypometabolism in the epileptogenic brain region, which is responsible for the onset of seizures. However, it appears that this hypometabolism extends beyond the epileptogenic focus and involves other brain regions in which epileptic activity spreads during seizures due to neighboring relationships and functional connections [3,6,16,18]. In studies using quantitative analysis methods, cortical asymmetry values ​​of temporal lobe were used to determine lateralization in brain FDG PET images [3,5,9,11,20]. In these studies, the asymmetry between temporal lobes showed better results in terms of lateralization compared with numerical analyses performed with global normalization [5,11]. The results obtained in the voxel-based statistical evaluations vary according to the statistical threshold values ​​used and there is no threshold value that can be applied as a standard. This may give priority to the use of AI calculation for lateralization. In addition, AI calculation eliminates the need for a regional reference selection and count normalization by definition. The aim of this study was to use asymmetry findings of mesial TLE patients in other brain regions, which are known to be affected by neighboring relationships or dynamic connections, besides mesial temporal structures for lateralization purposes [3,6,16,18]. This approach is expected to contribute to the lateralization of the epileptogenic temporal lobe. In fact, in this study, the model obtained by LMT, which uses asymmetry values ​​of multiple regions (hippocampus and temporal pole of the middle temporal gyrus) together, produced better results when compared with the model of J48 algorithm which is based on data of a single region (hippocampus), although the difference is not statistically significant.

The inclusion of TLE patients with Engel I outcome, according to at least 2-year follow-up results is a strong aspect of the study in terms of the reliability of lateralization results. Compared with similar studies in the literature, the number of patients is acceptable. However, in data mining applications, during the supervised learning phase, a large number of marked data is required to be presented to the algorithm. Therefore, the number of patients in the study may not be sufficient to establish a generalizable classification method with data mining. For this reason, k-fold cross-validation method is used to increase the knowledge about the behavior of algorithms used [31]. In addition, the test data used in the study can be classified with 100% accuracy in visual evaluation. This high accuracy rate, which contradicts with the 60% to 90% correct lateralization rates reported in the literature [37], may be related to the strong PET experience of the readers. In addition, the characteristic of the included patient group may be a bias for the study to give high accuracy rates. 

In conclusion, this study showed that data mining methods using regional metabolic asymmetry values ​​obtained from interictal brain FDG PET images in mesial TLE patients have high accuracy in the lateralization of epileptogenic temporal lobe. Therefore, data mining applications can contribute to the process of visual evaluation of brain FDG PET images by the reader. Investigation of different data mining methods by using series with a higher number and a broader spectrum of epilepsy patients may be helpful in guiding the use of this method in routine clinical applications.

## References

[ref1] (2017). Presurgical focus localization in epilepsy: PET and SPECT. Seminars in Nuclear Medicine.

[ref2] (2017). The role of radionuclide imaging in epilepsy, Part 1: Sporadic temporal and extratemporal lobe epilepsy. Journal of Nuclear Medicine Technology.

[ref3] (2000). Statistical parametric mapping of regional glucose metabolism in mesial temporal lobe epilepsy. Neuroimage.

[ref4] (2007). The contribution of 18F-FDG PET in preoperative epilepsy surgery evaluation for patients with temporal lobe epilepsy: A meta-analysis. Seizure-European Journal of Epilepsy.

[ref5] (2012). Usefulness of extent analysis for statistical parametric mapping with asymmetry index using inter-ictal FGD-PET in mesial temporal lobe epilepsy. Annals of Nuclear Medicine.

[ref6] (2012). Voxel-based comparison of preoperative FDG-PET between mesial temporal lobe epilepsy patients with and without postoperative seizure-free outcomes. Annals of Nuclear Medicine.

[ref7] (2014). Long-term epilepsy surgery outcomes in patients with PET-positive, MRI-negative temporal lobe epilepsy. Epilepsy & Behavior.

[ref8] (2015). Surgical outcome in patients with MRI-negative, PET-positive temporal lobe epilepsy. Seizure-European Journal of Epilepsy.

[ref9] (2016). Novel assessment of global metabolism by F-18-FDG-PET for localizing affected lobe in temporal lobe epilepsy. Nuclear Medicine Communications.

[ref10] (2010). Epilepsy duration impacts on brain glucose metabolism in temporal lobe epilepsy: Results of voxel-based mapping. Epilepsy & Behavior.

[ref11] (2008). Annals Voxel- and ROI-based statistical analyses of PET parameters for guidance in the surgical treatment of intractable mesial temporal lobe epilepsy. Nuclear Medicine.

[ref12] (2014). Can we increase the yield of FDG-PET in the preoperative work-up for epilepsy surgery. Epilepsy Research.

[ref13] (2014). The utility of 18F-fluorodeoxyglucose PET (FDG PET) in epilepsy surgery. Epilepsy Research.

[ref14] (2010). Objective detection of epileptic foci by 18F-FDG PET in children undergoing epilepsy surgery. Journal of Nuclear Medicine.

[ref15] (1998). Objective method for localization of cortical asymmetries using positron emission tomography to aid surgical resection of epileptic foci. Computer Aided Surgery.

[ref16] (2016). Determinants of brain metabolism changes in mesial temporal lobe epilepsy. Epilepsia.

[ref17] (2017). (18)F-FDG-PET patterns of surgical success and failure in mesial temporal lobe epilepsy. Neurology.

[ref18] (2012). Relationship between preoperative hypometabolism and surgical outcome in neocortical epilepsy surgery. Epilepsia.

[ref19] (2006). Correlations of interictal FDG-PET metabolism and ictal SPECT perfusion changes in human temporal lobe epilepsy with hippocampal sclerosis. Neuroimage.

[ref20] (2018). Temporal lobe asymmetry in FDG-PET uptake predicts neuropsychological and seizure outcomes after temporal lobectomy. Epilepsy & Behavior.

[ref21] (2019). Artificial intelligence in nuclear medicine. Journal of Nuclear Medicine.

[ref22] (2009). Zeileis A. Open-source machine learning: R meets Weka. Computational Statistics.

[ref23] (2005). Logistic model trees. Machine Learning.

[ref24] (2000). Localization of epileptogenic zones in F-18 FDG brain PET of patients with temporal lobe epilepsy using artificial neural network. IEEE Transactions on Medical Imaging.

[ref25] (2018). Global temporal lobe asymmetry as a semi-quantitative imaging biomarker for temporal lobe epilepsy lateralization: A machine learning classification study. Hellenic Journal of Nuclear Medicine.

[ref26] (2001). Proposal for a new classification of outcome with respect to epileptic seizures following epilepsy surgery. ILAE Commission Report.

[ref27] (2009). Computational anatomy with the SPM software. Magnetic Resonance Imaging.

[ref28] (2003). An automated method for neuroanatomic and cytoarchitectonic atlas-based interrogation of fMRI data sets. Neuroimage.

[ref29] (2000). Automated Talairach atlas labels for functional brain mapping. Human Brain Mapping.

[ref30] (2002). Automated anatomical labeling of activations in SPM using a macroscopic anatomical parcellation of the MNI MRI single-subject brain. Neuroimage.

[ref31] (2013). Principles of data mining.

[ref32] (2020). Yöntemleri. 4.

[ref33] (C4). 5: Programs for Machine Learning.

[ref34] (2014). Validation of an optimized SPM procedure for FDG-PET in dementia diagnosis in a clinical setting. NeuroImage: Clinical.

[ref35] (2017). Tomography/CT Improves Detection of the Epileptogenic Zone in Patients with Pharmacoresistant Epilepsy. Automated Online Quantification Method for F-.

[ref36] (1999). studies in patients with extratemporal and temporal epilepsy: Evaluation of an observer-independent analysis. Journal of Nuclear Medicine.

